# FIB-4 Regression With Direct-Acting Antiviral Therapy in Patients With Hepatitis C Infection: A Safety-Net Hospital Experience

**DOI:** 10.3389/fmed.2020.00359

**Published:** 2020-07-22

**Authors:** Sara Ghoneim, Muhammad Umer Butt, Sophie Trujillo, Imad Asaad

**Affiliations:** ^1^Department of Internal Medicine, Case Western Reserve University at MetroHealth Medical Center, Cleveland, OH, United States; ^2^Division of Cardiology, Case Western Reserve University at MetroHealth Medical Center, Cleveland, OH, United States; ^3^Division of Gastroenterology, Case Western Reserve University at MetroHealth Medical Center, Cleveland, OH, United States

**Keywords:** chronic hepatitis C infection, direct-acting antivirals, FIB-4 score, non-alcoholic fatty liver disease, both alcoholic fatty liver disease, diabetes mellitus, alcohol use disorder, hepatocellular carcinoma

## Abstract

**Background:** Liver fibrosis stage determines the risk of morbidity and mortality from chronic hepatitis C virus (HCV) infection. The majority of HCV-infected patients are underserved and have other comorbid conditions that lead to more progressive liver disease such as cirrhosis and hepatocellular carcinoma. Safety net hospitals are the prime location to treat these patients. Direct acting antiviral (DAA) agents are highly effective in virus eradication.

**Aim:** We aimed to evaluate the effect of treatment with DAAs on FIB-4 index.

**Methods:** We identified 343 patients who initiated HCV treatment with DAAs from 2016 to 2018 and achieved a sustained virologic response (SVR) in Metrohealth Medical Center, a safety net hospital system. We compared the severity of hepatic fibrosis before and 1 year after SVR was attained. We evaluated whether the presence of other comorbid conditions influenced liver fibrosis regression. All analyses were performed using SAS software.

**Results:** There was a statistically significant drop in mean FIB-4 score from baseline to post-SVR (3.47 ± 2.84 vs. 2.28 ± 1.60, *P* < 0.001). One hundred seventeen patients had baseline FIB-4 scores ≥3.25, 56% had FIB-4 scores <3.25 after SVR. Alcohol use disorder was associated with a higher baseline FIB-4 score compared to low level drinking (3.85 ± 0.20 vs. 3.15 ± 0.16). These patients showed greater improvement in FIB-4 scores after treatment when compared to those without alcohol use disorder (1.44 ± 0.15 vs. 0.97 ± 0.13, *P* = 0.02).

**Conclusion:** FIB-4 index is a useful non-invasive tool for monitoring fibrosis regression after antiviral therapy. Patients with a history of alcohol abuse had the greatest reduction in FIB-4 score post-SVR.

## Introduction

Chronic hepatitis C infection is a major health problem. There are 71 million individuals infected with hepatitis C virus (HCV) worldwide with 55–85% progressing to chronic liver disease (1). Approximately 15–30% are at risk of developing cirrhosis within 20 years of diagnosis (2). Hepatocellular carcinoma (HCC) is the most feared complication of HCV infection and is estimated to occur in 1–3% of patients over 30 years (3). The risk of HCC increases with progressive fibrosis, with most cases seen among patients with advanced fibrosis and/or cirrhosis making it essential for HCC surveillance to be included in current guideline recommendations (2).

Numerous extrahepatic manifestations have been reported suggesting HCV to be a systemic disease rather than just a liver disorder. Chronic hepatitis C infection has been shown to be associated with diabetes mellitus and fatty liver disease. A meta-analysis that included 48 studies showed diabetes mellitus to be strongly associated with cirrhosis [pooled odds ratio (OR) 2.5, 95% confidence interval, 1.8–3.5] (2). Moreover, diabetes mellitus is frequently seen in HCV-infected patients. One underlying mechanism may involve the immunologic imbalance induced by viral activation that leads to impaired glucose uptake into cells (4). Insulin resistance ensues and promotes abnormal fatty acid metabolism and accumulation into hepatocytes (5). Conversely, severe fibrosis and cirrhosis are able to induce glucose metabolism impairment.

Just recently has heavy alcohol consumption been shown to exert a synergistic effect with HCV on the progression of hepatic fibrosis (6). Significant alcohol consumption, defined as >60 g per day, in addition to HCV was associated with a 2.33 risk of cirrhosis compared to no or low-quantity alcohol consumption. Former drinkers who quit 1–10 years prior also had a higher risk of cirrhosis than current drinkers (7). A newly coined term, BAFLD defined patients with both significant alcohol consumption and non-alcoholic fatty liver disease (NAFLD) (8). Compared to patients with NAFLD, those with BAFLD had higher alanine aminotransferase (ALT), aspartate aminotransferase (AST), and lower platelet counts. They also had advanced fibrosis based on FIB-4 score ≥2.67 (8). Ekstedt et al. also found hepatic fibrosis to be accelerated in patients with hepatic steatosis who had consumed moderate amounts of alcohol (9).

Treatment to eradicate HCV infection provides benefits with respect to hepatic and extra-hepatic morbidity and mortality. In cases of advanced fibrosis, the benefits of direct acting antivirals (DAA) are observed after eradication of the virus. Achieving sustained viral response (SVR) is associated with a reduction in the rate of fibrosis progression, an overall decrease in the risk of HCC and liver-related mortality (10). Therefore, identifying patients with fibrosis is necessary to prioritize treatment. Ideally all patients with chronic hepatitis C infection should be treated, however the huge economic burden on healthcare makes this goal difficult to achieve. Evaluating the severity of liver fibrosis as suggested by American Association for the Study of Liver Diseases (AASLD) is essential prior to initiating therapy and provides a balanced approach to treating these complex and often heterogeneous group of patients (11). The two most widely used non-invasive tools for identifying and staging hepatic fibrosis are transient elastography (TE) and FIB-4 index. Transient elastography measures liver stiffness by assessing the propagating velocity of shear waves through the hepatic parenchyma (12). The fibrosis-4 index (FIB-4) is one of the many serological testes developed to detect liver fibrosis in patients with chronic hepatitis C. A FIB-4 score <1.45 has a negative predictive value of 95.7% to exclude severe fibrosis with a sensitivity of 74.3%. A FIB-4 score ≥3.25 has a 97% specificity and a positive predictive value of 65% of advanced fibrosis (13, 14). A recent study comparing various risk scores and elastography (magnetic resonance and TE) against liver histology showed FIB-4 to better than other indices such as BARD, aspartate aminotransferase (AST)-to-platelet ratio index (APRI), AST/ALT ratio, and as good as MRE for predicting advanced fibrosis in patients with NAFLD (15, 16). Currently, AASLD recommends the use FIB-4 score in the Simplified HCV Treatment Algorithm as a non-invasive pre-treatment assessment of liver fibrosis, using 3.25 as a cut-off for advanced fibrosis/cirrhosis (11).

Urban safety-net hospitals are the prime location for HCV treatment. Patients served by these systems are often below the national poverty level and are comprised of a unique mix of ethnic minorities. The application of non-invasive tools such as FIB-4 and TE in evaluating the severity of hepatic fibrosis before and after therapy may have an advantage and potential implications in the community. Also, whether factors such as heavy alcohol use, hepatic steatosis, or insulin resistance influence the magnitude of fibrosis regression after treatment is important to consider given the rising rates of metabolic syndrome in the United States. Our current study aimed to compare the severity of hepatic fibrosis before and after treatment with DAAs and to identify factors that may influence liver fibrosis regression in an American urban population followed in a safety-net hospital.

## Materials and Methods

### Data Source

We identified 399 adult patients with active chronic HCV infection seen in our liver clinic at Metrohealth Medical Center; a safety net health system, affiliated with Case Western Reserve University from 2016 to 2018. Patients were identified through ICD-9 and ICD-10 codes 070.54 and B18.2, respectively. After manual chart review, we excluded 56 patients who had a diagnosis of chronic HCV infection but were lost to follow up or were non-compliant with DAAs. All 343 subjects included in the study achieved SVR, defined as a serum HCV RNA viral load below the lower limit of detection performed at least 12 weeks after the end of HCV treatment (17).

### Baseline Characteristics Before Antiviral Treatment

We collected baseline data before antiviral treatment including age, sex, ethnicity, HCV genotype, and prior antiviral treatment experience. We extracted all laboratory tests before treatment and recorded the value of each test closest to the date of treatment initiation within the preceding 6 months as the baseline value. We determined the presence of insulin resistance (IR), type 2 diabetes (DM), NAFLD, alcohol use disorders by manual chart review for ICD-9 or ICD-10 codes recorded at least once before treatment. Insulin resistance was defined as a hemoglobulin (Hb) A1c of ≥5.7% and diabetes mellitus as A1c values ≥6.5%. Alcohol use disorder was defined as consumption of more than 2 drinks/day or 14 drinks/week and 3 drinks/day or 21 drinks per week for female and male, respectively. Patients with significant alcohol intake who quit within 1–10 years prior to treatment were recorded as those with alcohol use disorder and identified by the corresponding ICD code recorded. All patients were in remission prior to initiating DAAs. Patients with alcohol use disorder had serum ethanol levels obtained prior to initiating, and during treatment to confirm abstinence. When identifying patients with NAFLD, we followed the criteria established by AASLD which included: the presence of hepatic steatosis seen on imaging or histology, the lack of secondary causes of hepatic fat accumulation such as significant alcohol consumption, long-term use of steatogenic medications, or monogenic hereditary disorders (17). Liver ultrasound was used to identify patients with evidence of hepatic steatosis recorded prior to initiating treatment. An ultrasound probe was used to obtain the following images: sagittal view of the right lobe of the liver and right kidney, transverse view of the lateral segment of the liver and spleen, transverse view of the liver, and pancreas and of any focal areas of altered echotexture. Steatosis was defined when the echogenicity of the liver exceeded that of the renal cortex and spleen; there was increased attenuation of the ultrasound beam causing posterior darkness and loss of definition of the diaphragm and when the intrahepatic architectural detail was difficult to visualize. Patients with BAFLD were identified as those with alcohol use disorder and NAFLD documented prior to initiating treatment.

### Fibrosis-4 Scores Before Treatment and After Treatment

We used baseline laboratory tests within 6 months before the initiation of DAAs to calculate the baseline FIB-4 score as follows:

$$\begin{array}{l} \frac{\left(Age\;\times \;AST\right)}{\left(Platelet\;count\;\times \;ALT\right)} \end{array}$$

We also extracted all laboratory tests 1 year after SVR was achieved and used them in combination to the patients' age to calculate their post-SVR FIB-4 scores. We categorized patients based on the following FIB-4 scores: Baseline FIB-4 ≥ 3.25 and after treatment FIB-4 <3.25. We were also interested in evaluating whether regression in FIB-4 score was affected by demographical data, alcohol use, insulin resistance/diabetes mellitus, the presence of NAFLD, or BAFLD.

### Statistical Analysis

Data on demographics, comorbid conditions, liver biochemical tests at baseline, and after DAA-induced SVR and clinical outcomes were collected as previously described. Change in FIB-4 scores before and post-SVR was primary outcome. FIB-4 score >3.25 was considered as advanced fibrosis and improvement in fibrosis was considered if it decreased to <3.25 following treatment. We presented categorical data as frequencies and percentages of the total. We cross-tabulated categorical variables with Chi-square test to determine whether the observed distribution fitted the expected distribution when the cell size was sufficient. When the cell size was not enough Fisher's exact test was used. For continuous covariates, dependent samples *t*-test or Wilcoxon signed rank test were applied.

In prespecified subgroup analysis we ran linear mixed-model analysis of variance (ANOVA) models with restricted maximum likelihood estimation. Models controlled for the between-subject nature variance of by including random effects for patients. Treatment was included as a fixed, between-subjects factor to models FIB-4 score. For subgroup analysis each specified variable was included in the model as fixed effect interacting with main effect of treatment. We captured the within subject variation directly by modeling the variance-covariance matrix of the residuals. Compound symmetry variance-covariance was retained as it did not show improvement in fit compared to the unstructured best-fitting variance-covariance matrix structure, [−2RLL (restricted log likelihood) = 2868.1].

Statistical analysis was performed with SPSS version 26.0 (SPSS Inc, Chicago, IL) and SAS version 9.4 (SAS Inst., Cary, NC). Significance was defined as the 2-tailed value of *p* < 0.05.

### Regulatory Approvals

This study (IRB18-00733) was approved by the Case Western Reserve University at Metrohealth Medical Center Institutional Review Board.

## Results

### Baseline Characteristics of Patients

In our cohort of 343 patients, 208 (60.6%) were males, 191 (55.7%) were African Americans, 124 (36.1%) were Caucasian and 24 (7%) were Hispanics. The mean age was 60.52 ± 8.48 years (Table 1). The majority of patients were HCV genotype 1a (65.6%) positive. A total of 291 patients (84.8%) were treatment naïve. The majority of patients were treated with Harvoni®, 162 (47.7%), 70 (20.41%) with Zepatier®, and 50 (15%) with Epclusa®. Approximately 43% of patients had a history excessive alcohol consumption. The mean Hb A1c of patients before treatment with DAAs was 6.17 ± 1.83 gm/dL. Approximately 23% were diagnosed with BAFLD. A total of 161 patients had a diagnosis of NAFLD prior to treatment with DAAs.

**Table 1:** Baseline characteristics.

**Baseline characteristic**	**Total cohort (*n* = 343)**
**Age, y (mean, SD)**	60.52 ± 8.48
**Gender**	
Male	208
Female	135
**Race**	
African American	191 (55.69%)
Caucasian	124 (36.15%)
Hispanic	24 (7%)
Others	4 (1.16%)
**Treatment naïve**	291 (84.84%)
**NAFLD**	161 (46.94%)
**BAFLD**	74 (21.57%)
**Genotype**	
IA	225 (65.60%)
IB	56 (16.33%)
Other	62 (18.07)
**Treatment**	
Harvoni^®^	162 (47.23%)
Harvoni® +Ribavirin	22 (6.41%)
Zepatier®	70 (20.41%)
Epclusa®	50 (15%)
Other	34 (16.04%)
**IR/DM**	142 (41.40%)
**Hb A1c**	6.17 ± 1.83 gm/dL
**Alcohol use disorder**	146 (42.57%)

### FIB-4 Score and Laboratory Tests at Baseline and After Treatment

Table 2 shows the mean values for aspartate aminotransferase (AST), alanine aminotransferase (ALT), platelets at baseline, and after achieving SVR. A total of 110 (32.07%) patients were thrombocytopenic before treatment and this number was reduced to 84 (24.29%) after treatment. Approximately 75% (82/110) of them had baseline FIB-4 ≥ 3.25 and 43 (52%) had FIB-4 <3.25 after treatment. There was statistically significant increase in platelet counts after treatment (181.15 ± 71.87 vs. 194.56 71.86, *P* < 0.001).

**Table 2:** Primary and Secondary outcomes before and after treatment.

**Characteristic**	**Pre-treatment Fib4 score**	**Post-SVR Fib4 score**	**Degree of change (Pre-treatment -post-treatment) mean (CI)**	***P-*value**
AST	67.23 ± 49.4	28.50 ± 13.30	−38.73 (33.84–43.61)	<0.001
ALT	68.86 ± 6 6.85	23.40 ± 12.75	−45.45 (33.85–43.61)	<0.001
Platelets	181.15 ± 71.87	194.56 ± 71.86	+13.40 (−17.93 to −8.89)	<0.001
FIB-4	3.45 ± 2.84	2.28 ± 1.60	−1.17 (0.97–1.36)	<0.001

### The Drop in FIB-4 Score From ≥ 3.25 Pre-treatment to <3.25 After Treatment

**Figure 1:**
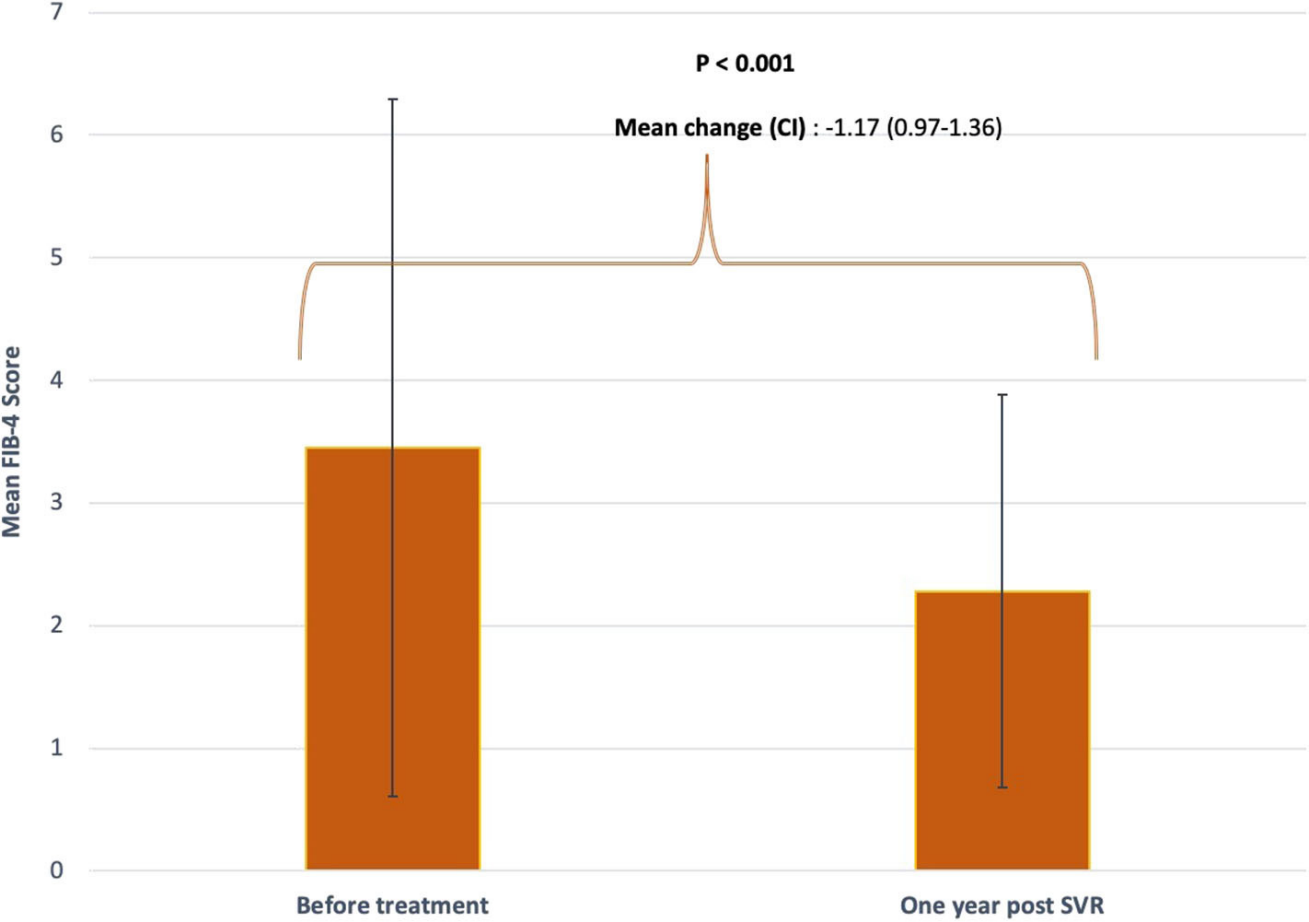
Mean FIB-4 score before and after treatment. FIB-4 scores were obtained at baseline prior to initiating DAA and 1 year after achieving SVR. Standard deviations are represented by vertical lines.

There was a statistically significant drop in mean FIB-4 score from FIB-4 ≥3.25 to <3.25 after DAA-induced SVR (3.47 ± 2.84 vs. 2.28 ± 1.60, *P* < 0.001) (Figure 1). Out of 343 patients, 117 (34%) had baseline FIB-4 ≥3.25, 67 of which attained FIB-4 <3.25 post-SVR (55%) while 50 (45%) had persistently elevated FIB-4 ≥ 3.25 even after treatment. A total of 226 (66%) patients had baseline FIB-4 <3.25. After treatment, this number increased to 287 subjects (83.7%).

Of note, 34% (117/343) of our cohort achieved FIB-4 <1.45 post treatment, indicating a high probability of low level fibrosis (F0-F-1 Metavir stages) (12, 13).

### Subgroup Analysis on FIB-4 Score Pre-treatment and After Treatment

**Figure 2:**
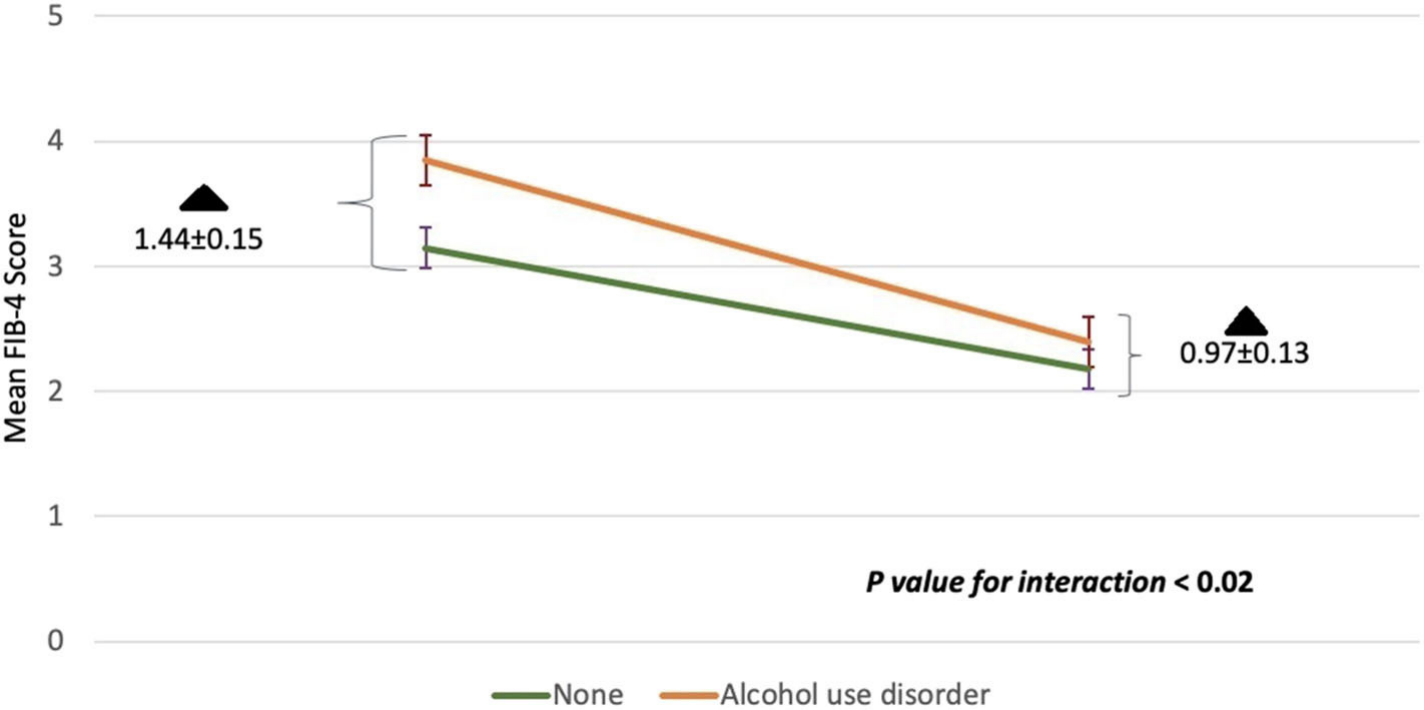
The interaction between alcohol use disorder with DAAs on mean FIB-4 scores before and after treatment. Baseline FIB-4 scores for the heavy alcohol use group or none are shown in the left corner. FIB-4 scores 1 year post-SVR are shown in the right corner. The rate of change in FIB-4 among those with alcohol use disorder was higher compared to those without (*p* < 0.02). The degree of change (black triangle) among both groups at the beginning of the study and at the end was also statistically significant (*p* < 0.02). Standard deviations are represented by vertical lines.

We sought to evaluate whether comorbid conditions frequently encountered in HCV patients influenced the change in FIB-4 index with treatment. The interaction of DAAs with study subgroups is summarized in Table 3. Across all subgroups, the mean aggregate baseline FIB-4 scores was >3.25, suggesting advanced fibrosis, except for: age groups younger than 60, absence of alcohol use disorder, and HCV genotypes other than 1a subgroups. The mean FIB-4 score after treatment in all subgroups was consistently <3.25. No statistically significant interaction between study subgroups and DAAs was observed except with alcohol use. Heavy alcohol consumption was associated with a statistically significant change in FIB-4 values pre-and post-treatment. Although heavy alcohol consumption was associated with a higher baseline FIB-4 score compared to low level drinking (3.85 ± 0.20 vs. 3.15 ± 0.16), these patients showed greater improvement in FIB-4 scores after treatment when compared to those without alcohol use disorder (1.44 ± 0.15 vs. 0.97 ± 0.13, *P* = 0.02) (Figure 2).

**Table 3:** Subgroup analysis difference in FIB-4 score before and post-treatment.

**Subgroups**	**Pre-treatment FIB-4 score**	**Post-treatment FIB-4 score**	**Degree of change (Pre-treatment -post-treatment)**	***P-*value for interaction**
**Age**				**0.28**
<60	3.05 ± 0.20	2.02 ± 0.20	1.04 ± 0.15	
≥60	3.71 ± 0.16	2.45 ± 0.16	1.25 ± 1.25	
**Gender**				**0.32**
Male	3.27 ± 0.16	2.19 ± 0.16	1.09 ± 0.12	
Female	3.71 ± 0.20	2.42 ± 0.2	1.29 ± 0.15	
**Race**				**0.64**
Caucasian	3.57 ± 0.21	2.34 ± 0.21	1.23 ± 0.16	
Non-caucasian	3.37 ± 0.16	2.24 ± 0.16	1.13 ± 0.12	
**NAFLD**				**0.63**
No	3.60 ± 0.17	2.39 ± 0.17	1.21 ± 0.13	
Yes	3.27 ± 0.18	2.15 ± 0.18	1.11 ± 0.14	
**BAFLD**				**0.07**
No	3.36 ± 0.14	2.28 ± 0.14	1.10 ± 0.11	
Yes	3.76 ± 0.27	2.26 ± 0.27	1.51 ± 0.21	
**Genotype**				**0.26**
Non IA	3.10 ± 0.21	2.07 ± 0.21	1.02 ± 0.17	
IA	3.63 ± 0.15	2.38 ± 0.15	1.25 ± 0.12	
**Alcohol use disorder**				**0.02**
No	3.15 ± 0.16	2.18 ± 0.16	0.97 ± 0.13	
Yes	3.85 ± 0.20	2.41 ± 0.20	1.44 ± 0.15	
**IR/DM**				**0.17**
No	3.58 ± 0.16	2.30 ± 0.16	1.28 ± 0.13	
Yes	3.25 ± 0.19	2.24 ± 0.19	1.01 ± 0.15	

## Discussion

Most HCV patients undergo antiviral treatment with DAAs and are expected to attain SVR. A DAA-induced SVR reduces the patient's risk of cirrhosis, HCC and extrahepatic manifestations of HCV infection (18). Our liver clinic treats the most vulnerable populations who are not only disproportionately affected by HCV, but have multiple comorbid medical conditions associated with this infection. As previously mentioned, IR and DM are associated with hepatic steatosis development. Rapid progression of fibrosis to cirrhosis occurs in patients with HCV infection (19, 20). The presence of glucose abnormalities has also been shown to negatively influence the effects of HCV on the liver and its treatment (21). In our model, the presence of insulin resistance or diabetes did not modify the effect DAAs had on FIB-4 scores prior to and after SVR. This observation held true for patients with pre-treatment history of NAFLD or BAFLD. History of heavy alcohol consumption was the only factor with direct impact on FIB-4 index. The mean drop in FIB-4 score was also significantly higher in this group than in those without alcohol abuse. The reported prevalence of alcohol abuse in HCV patients is estimated to be between 14–36% (19–24). Our results demonstrated that 42% of subjects had a history of alcohol abuse, well above what is currently estimated. Recent studies have shown heavy alcohol consumption to be associated with an least 34% increase in the rate of fibrosis progression (22). It was also associated with an increased risk of HCC compared to those with HCV infection alone (9). Previously it was shown that former drinkers who had stopped 1–10 years previously had a higher risk of HCC than current drinkers and that the risk of HCC increased with increasing level of alcohol intake irrespective of duration of consumption or age to start (9). Our findings may therefore have important implications. First, significant fibrosis regression can still be achieved with DAAs irrespective of whether metabolic syndrome components exist or not. Second, it appears that patients who would benefit most from DAA therapy are those with alcohol use disorder and more advanced fibrosis. Currently there is no data on the interaction of alcohol use and DAAs on liver fibrosis regression. Experimental and clinical studies are warranted to improve our knowledge on the consequence of this interaction.

Briefly, over half of our patients had baseline FIB-4 ≥3.25 with ~56% achieving FIB-4 <3.25 while 45% had persistently elevated FIB-4 scores despite achieving SVR. Although FIB-4 ≥3.25 was developed as a measure of advanced fibrosis, it is emerging as a convenient marker for HCC risk in patients with chronic HCV infection, even without a pre-treatment diagnosis of cirrhosis (25, 26). A drop in FIB-4 from ≥3.25 before treatment to <3.25 after treatment was shown to be consistently associated with a reduced risk of HCC, whereas an increase in FIB-4 ≥3.25 post-SVR was associated with a substantial increase in HCC risk per year (27). Safety net hospitals must cope with limited resources and a patient population with complex medical needs. These factors highlight the potential utility of non-invasive fibrosis assessments such as FIB-4 index in identifying patients who might benefit from HCC surveillance once SVR is achieved. Current guidelines recommend patients with cirrhosis to continue HCC surveillance indefinitely even after SVR (28–30). However, it's unclear as to whether patients with advanced fibrosis but not cirrhosis should undergo HCC surveillance or not after SVR. Accordingly, these patients should be offered HCC screening if their pre-treatment FIB-4 score is ≥3.25 and especially if it remains ≥3.25 after SVR.

Thrombocytopenia was observed in 34% of our patients prior to treatment and this was reduced to only 24% after SVR. Common factors affecting platelet count in patients with chronic liver disease include hepatic fibrosis, necroinflammation, and thrombopoietin (31). Liver fibrosis stage has been shown to be inversely associated with platelet count in patients with chronic hepatitis B and chronic liver disease (32). Platelets interact with hepatic sinusoidal endothelium while circulating the injured liver and recruit proinflammatory cells and proteins. This activity results in a self-perpetuating cycle of platelet and leukocyte accumulation resulting in further hepatocellular injury (33). One study showed hepatic necroinflammatory activity to correlate with low platelet counts in chronic hepatitis C disease (34). Our study demonstrated reduction in hepatic necroinflammation as evidenced by increase in platelet count after SVR. This observation supports the hypothesis that platelet count increases as a consequence of reduction in necroinflammation.

Our study had several limitations that should be addressed. First, it was retrospective in design and relied exclusively on electronic medical record data that was recorded during routine clinical care. Second, our analyses were performed using FIB-4 index, a non-invasive non-imaging based assessment of fibrosis likelihood. No liver biopsies were obtained to examine the temporal changes occurring in FIB-4 values before and after treatment. However, liver biopsy is an invasive procedure, and its use cannot be widely applied to clinical practice, especially to safety-net hospitals were resources are extremely limited. Third, our study was conducted at a single center where only 343 patients were enrolled. Larger studies comparing FIB-4 index against image-based or invasive methods of fibrosis assessment would clarify the performance characteristic of this simple non-invasive approach to characterizing fibrosis risk in the underserved populations. Despite these limitations, our study was able to demonstrate the potential usefulness of FIB-4 index for identifying and monitoring HCV patients with advanced fibrosis receiving DAA therapy in the community.

## Data Availability Statement

All datasets generated for this study are included in the article/supplementary material.

## Ethics Statement

The studies involving human participants were reviewed and approved by Case Western Reserve/Metrohealth Medical Center IRB approved this study. The patients/participants provided their written informed consent to participate in this study.

## Author Contributions

SG was the guarantor and designed the study. All authors participated in the acquisition, analysis and interpretation of the data, drafted the initial manuscript, and revised the article critically for important intellectual content.

## Conflict of Interest

The authors declare that the research was conducted in the absence of any commercial or financial relationships that could be construed as a potential conflict of interest.
